# Cell Viability Assessment Using Fluorescence Vital Dyes and Confocal Microscopy in Evaluating Freezing and Thawing Protocols Used in Cryopreservation of Allogeneic Venous Grafts

**DOI:** 10.3390/ijms221910653

**Published:** 2021-09-30

**Authors:** Pavel Měřička, Libor Janoušek, Aleš Benda, Radka Lainková, Ján Sabó, Markéta Dalecká, Petra Prokšová, Myroslav Salmay, Rudolf Špunda, Ondřej Pecha, Miroslava Jandová, Jiří Gregor, Lubomír Štěrba, Miroslav Špaček, Jaroslav Lindner

**Affiliations:** 1 Tissue Bank, University Hospital, 500 05 Hradec Králové, Czech Republic; pavel.mericka@fnhk.cz (P.M.); jandomir@fnhk.cz (M.J.); jiri.gregor@fnhk.cz (J.G.); lubomir.sterba@fnhk.cz (L.Š.); 2 Department of Transplantation Surgery, Institute for Clinical and Experimental Medicine, 140 21 Prague, Czech Republic; lija@medicon.cz; 3 Imaging Methods Core Facility at BIOCEV, Faculty of Science, Charles University, 252 50 Prague, Czech Republic; ales.benda@natur.cuni.cz (A.B.); jan.sabo@natur.cuni.cz (J.S.); marketa.dalecka@natur.cuni.cz (M.D.); petra.proksova@natur.cuni.cz (P.P.); 4 2nd Department of Surgery–Department of Cardiovascular Surgery, 1st Medical Faculty, Charles University and General University Hospital, 128 08 Prague, Czech Republic; radka.lainkova@vfn.cz (R.L.); myroslav.salmay@vfn.cz (M.S.); rudolf.spunda@vfn.cz (R.Š.); jaroslav.lindner@vfn.cz (J.L.); 5 Department of Cell Biology, Charles University, Viničná 7, 128 00 Prague, Czech Republic; 6 Technology Centre of the Czech Academy of Sciences, 160 00 Prague, Czech Republic; onpecha@seznam.cz; 7 Department of Anatomy, Histology and Embryology Medical Faculty in Hradec Králové, Charles University, 500 03 Hradec Králové, Czech Republic

**Keywords:** cell viability, vascular allograft, fluorescence vital dyes, confocal microscopy, cryopreservation, thawing method

## Abstract

The authors present their contribution to the improvement of methods suitable for the detection of the freezing and thawing damage of cells of cryopreserved venous grafts used for lower limb revascularization procedures. They studied the post-thaw viability of cells of the wall of cryopreserved venous grafts (CVG) immediately after thawing and after 24 and 48 h culture at +37 °C in two groups of six CVG selected randomly for slow thawing in the refrigerator and rapid thawing in a water bath at +37 °C. The grafts were collected from multi-organ and tissue brain-dead donors, cryopreserved, and stored in a liquid nitrogen vapor phase for five years. The viability was assessed from tissue slices obtained by perpendicular and longitudinal cuts of the thawed graft samples using in situ staining with fluorescence vital dyes. The mean and median immediate post-thaw viability values above 70% were found in using both thawing protocols and both types of cutting. The statistically significant decline in viability after the 48-h culture was observed only when using the slow thawing protocol and perpendicular cutting. The possible explanation might be the “solution effect damage” during slow thawing, which caused a gentle reduction in the graft cellularity. The possible influence of this phenomenon on the immunogenicity of CVG should be the subject of further investigations.

## 1. Introduction

The authors present their contribution to the improvement of methods suitable for detection of the freezing and thawing damage of cells of cryopreserved venous grafts used for lower limb revascularization procedures. Cell viability assays are standard tools for the detection of this damage. In freezing cell suspensions, membrane integrity testing by vital dye exclusion is usually combined with the assessment of cell purity, use of flow cytometry, and testing the cell repopulation potency [1,2,3,4]. Assessment of the viability of cryopreserved tissues requires the application of more sophisticated methods. One approach is based on the isolation of cells by tissue digestion, followed by the application of the earlier mentioned cell viability and phenotyping assays. Another approach is based on in situ vital staining of cells [5]. Alternatively, different metabolic assays like MTT tests or the glucose uptake test are used [2]. Innovative approaches to cell viability assessment consider not only the results of tests performed immediately after thawing, but also the changes in the post-thaw cell viability through time [6,7,8]. This approach enables the detection of cryopreservation and storage induced apoptosis [9,10,11] and/or the delayed onset of cell death, leading to cell necrosis, apoptosis, or a combination of both [2,3,11]. After clinical application of thawed cell suspensions such as in hematopoietic progenitor cell (HPC) transplantation, certain cell types can become deficient, which can cause delayed engraftments or even non-engraftments or post transplantation anemia or thrombocytopenia [3,12,13,14]. In the case of solid tissue transplantation, the graft immunogenicity and/or the intensity of the graft rejection can be modified [2,15].

The aim of this study was to compare the post-thaw viability of cells of the wall of cryopreserved veins after two different thawing protocols, using in situ staining with fluorescence vital dyes, following the method of Johnson and Rabinovitch [5]. The viability was assessed immediately after thawing and a 24- and 48-h culture in the CO_2_ incubator at +37 °C. The study was part of the obligatory cryopreservation and storage process validation required by Czech legislation and international recommendations.

## 2. Results

### 2.1. Immediate Post-Thaw Cell Viability 

The basic descriptive statistics data are summarized in Table 1. High viability values, mean and median above 70%, were found in using both thawing protocols and both types of cuts.

**Figure 1:**
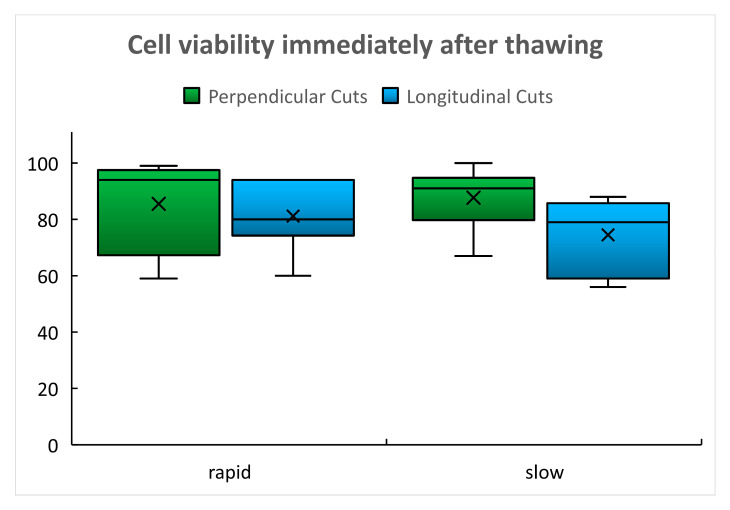
Immediate post-thaw cell viability (%).

The immediate post-thaw viability data revealed normal distribution and equality of variances after both thawing protocols. After slow thawing, slightly lower viability was found in LC (Table 1, Figure 1), but the difference was still not significant (*p* = 0.0996). After rapid thawing, there was no statistically significant difference between the viability assessed in LC and in PC (*p* = 0.623) (Table 1, Figure 1) (see Supplementary Materials).

### 2.2. Evaluation of Changes of the Post-Thaw Cell Viability in Time 

#### 2.2.1. Slow Thawing Protocol 

**Figure 2:**
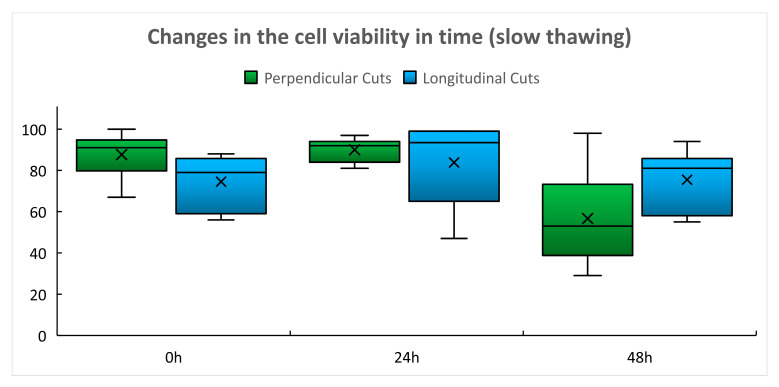
Changes of the post-thaw cell viability in time—slow thawing (%).

In the group of slowly thawed grafts, the mean cell viability assessed after 24 h of culture (Table 2, Figure 2) was practically identical as immediately after thawing. Decline in the cell viability in slices obtained by PC occurred after 48 h of culture (Table 2 and Figure 2, green box-plots). This difference was statistically significant after G–G correction performed because of the inequality of variances (48 h vs. 0 h, *p* = 0.014; 48 h vs. 24 h, *p* = 009). The mean cell viability in slices obtained by LC (Table 2, Figure 2, blue box plots) remained practically unchanged and globally statistically insignificant (*p* 0.4222) during the whole culture period.

#### 2.2.2. Rapid Thawing Protocol

**Figure 3:**
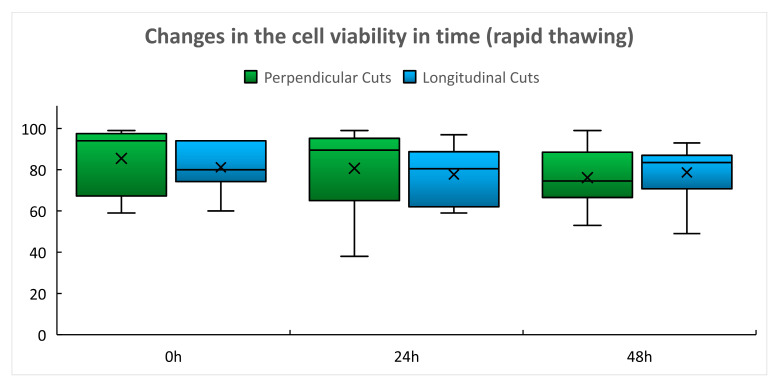
Changes in the post-thaw cell viability in time—rapid thawing (%).

In the group of rapidly thawed grafts, only minimal, statistically insignificant changes of cell viability with time were found in both types of cuts (Table 3, Figure 3). Global *p* value for perpendicular cuts was 0.6415 and 0.6718 for longitudinal cuts.

## 3. Discussion

Cryopreservation protocols leading to good post-thaw cell viability are still regarded as the gold standard for the preservation of cardiovascular tissues regardless of the fact that post-transplantation immunosuppressive therapy is necessary to prevent rapid deterioration of the implanted graft [16,17,18,19]. The presented results showed high immediate post-thaw cell viability in using both thawing protocols (Table 1). Undoubtedly, this is the result of proper control of pre-freezing conditions (avoiding the long warm pre-harvest ischemia by tissue collection within a multiple organ and tissue harvest, use of the organ preservation solution for storage of collected tissue during transport, and early processing of the collected tissue in the TE) [20] as well as of the efficient cryoprotection using 10% dimethyl sulfoxide (DMSO) and safe 3–5 year storage in the vapor phase of liquid nitrogen at a temperature below −160 °C [20]. Use of controlled-rate slow cooling, which is expected to form a relatively stable ice structure, may also contribute to these results as devitrification is less likely to occur during storage and thawing than in the case of using vitrification protocols [21]. The post-thaw cell viability was relatively stable during the 48 h culture of tissue slices, the interval in which the delayed onset of cell death regularly occurs [7] (Table 2 and Table 3, Figure 2 and Figure 3). Only after using the slow thawing protocol was there a statistically significant decline in cell viability in slices obtained by perpendicular cutting after 48 h culture (Table 2, Figure 2). We considered the cell viability results obtained by perpendicular cutting to be a more reliable indicator of the success of cryopreservation of vascular grafts as in this case, the cells of all vascular wall layers were evaluated, while in the case of longitudinal cuts, we predominantly tested the viability of the endothelial layer that is in direct contact with the culture medium. A deeper decline in viability after slow thawing was not surprising as slow thawing leads to a longer exposure of cells to concentrated electrolyte solutions at sub-zero temperatures and the cells are damaged by the so called “solution effect”, which may cause immediate or delayed cell death (Table 2, Figure 2). While in freezing cell suspensions, the best post-thaw viability is achieved if a combination of slow cooling and rapid thawing is used [4,7,12], such protocols may not be optimal for tissue cryopreservation. The research group of David Pegg already proved in the 90 s that micro-fractures of the arterial wall caused by devitrification may occur during rapid thawing [22,23]. Microfractures in human arterial grafts were also described by Novotný [24]. In the clinical situation, such an event can cause early graft rupture of thawed arterial grafts [16,17,25,26]. Such events were not confirmed by us in the case of venous grafts [27]. Nevertheless, we regard the combination of slow cooling and the slow thawing protocol as a compromise between achieving the high post-thaw cell viability and avoiding the risk of damage to the structural integrity of the thawed vascular graft [27]. The delayed onset of cell death events may also be triggered by the use of DMSO, which still remains a dominant cryoprotectant in the cryopreservation of vascular tissues [27,28], regardless of reports pointing out its toxicity [29]. The current version of the European Union Tissue Establishment Product List [30] does not include, however, any cardiovascular tissue cryopreserved with cryoprotectants other than DMSO. It is probable that the event of delayed cell death observed in this experimental model may occur after transplantation of the thawed vascular grafts and may cause gentle reduction in the graft cellularity, leading to the lowering of its immunogenicity as observed in experiments with rats [18,19]. For this reason, the possible effect of the different freezing and thawing protocols on the immunogenicity of CVG should be the subject of further research.

## 4. Methods

### 4.1. Vascular Tissue Harvest and Cryopreservation

The study was performed in 12 CVG (10 great saphenous veins, two femoral veins) harvested from multiple organs and tissue brain -dead donors. This was performed by a surgical team of the Department of Transplantation Surgery of the Institute for Clinical and Experimental Medicine in Prague, and the grafts were sent to the Tissue Establishment of the University Hospital Hradec Králové—EU TE CODE CZ000427 (TE) in a pre-cooled (+4 °C) organ preservation solution (Celsior, Genzyme, The Netherlands) supplemented with gentamicin (Gentamicin Lek, LEK Pharmaceuticals, Ljubljana, Slovenia). Transport was performed by Meditrans Ltd., Prague, Czech Republic, a company fully licensed to transport organs and tissues for transplantation. Cryopreservation was commenced within 24 h after the harvest [20,31]. After input control in the TE, the grafts were processed in the clean room area of grade A with background B. Decontamination was performed by immersing the grafts into an antibiotic solution following a modified method by van Kats [32]. After removal of the remnants of antibiotics by washing, the vessels were put into double sterile disposable plastic bags (Eva Bags, Maco Biotech, Mouvaux, France) containing 50 mL of pre-cooled 6% solution of hydroxyethyl starch, m.w. 130,00 Da (Voluven 6%, Fresenius Kabi, Bad Hamburg, Germany) that was mixed with an equal volume of the pre-cooled cryoprotective solution containing 20% dimethyl sulfoxide (WAK Chemie Medical, GmbH., Steinbach, Germany). The samples for the bacteriological and mycological tests were taken from both the tissue collection and transport solution and from the cryopreservation bag. Then, the cryopreservation bags were heat sealed, closed into metal cassettes, and frozen by the rate of 1 K/min to −90 °C and −5 K/min to −150 °C [20]. Afterward, they were stored in the liquid nitrogen vapor phase at a temperature below −160 °C in the biological container KRYO CE 10 K, Taylor Wharton, Germany, GmbH, Mildstedt, Germany, with an automatic filling system and continuous temperature monitoring. The grafts selected for this study did not meet the criterion of sterility, but other criteria required for their release for clinical application [20] were met. The grafts were randomized for choosing thawing protocol No. 1, which is slow thawing in a refrigerator (+2 to +8 °C) for two hours as routinely used in clinical practice [20,33] (six grafts), and protocol No. 2, which is rapid thawing in a water bath at +37 °C (six grafts). Warming was stopped at the moment when the last remnants of ice disappeared.

### 4.2. Processing of the Graft Samples for Viability Assays

**Figure 4:**
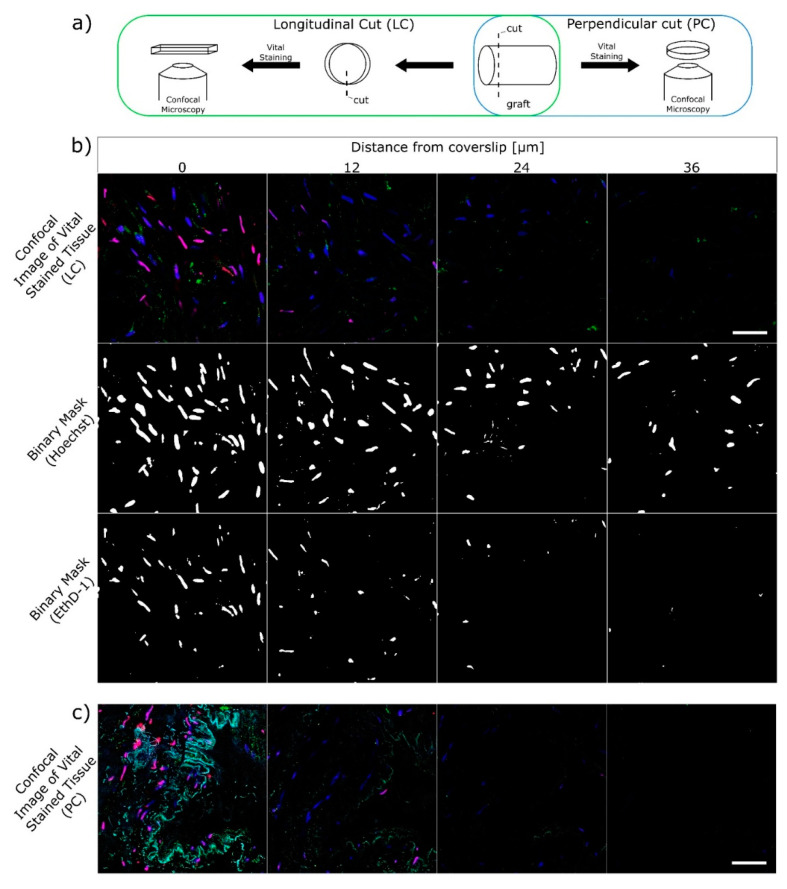
In situ staining of the thawed vascular graft sample with fluorescence vital dyes. (**a**) Scheme of the longitudinal (LC) and perpendicular (PC) cuts preparation. (**b**) Confocal images in four Z-planes for LC and subsequent binary masks used for automatic evaluation of viability. (Top) All cells in blue (Hoechst), dead cells in red (EthD-1), and enzymatic activity in green (calcein). (Middle and bottom) Comparison of dead cell count (EthD-1 mask) and total cell count (Hoechst mask). Scale bar 50 μm. (**c**) Confocal images in color for PC. Note the internal cavity of the graft and majority of the cells on the “cut” side stained red. Scale bar 50 μm.

Immediately after thawing, the grafts were removed from the cryoprotective solution, placed into the pre-cooled (+4 °C) organ preservation solution (Custodiol CE, Dr.Franz Kohler Chemie, GmbH, Bensheim, Germany), and transported for viability assessment. Each graft was divided into three segments: one was used for immediate viability assessment, while the remaining segments were cultured in the CO_2_ incubator for 24 and 48 h in the FluoroBrite DMEM culture medium (Thermo Fisher Scientific, Waltham, MA, USA) with 10% fetal bovine serum (Sigma Aldrich, St. Louis, MO, USA). The tissue slices for imaging were obtained by perpendicular cuts (PC) forming circular segments and by longitudinal cuts (LC) forming stripes (Figure 4a). In situ vital staining was performed with the use of the following fluorescence dyes according to protocol 6, as described by Johnson and Rabinovich [5].

1. Substance Hoechst 33258 (blue in Figure 4b,c; Sigma Aldrich, St. Louis, MO, USA) Nuclear stain that permeates cells regardless of the membrane status. Excitation 405 nm, emission 420–475 nm.
2. Acetoxymethyl calcein (green in Figure 4b,c; Thermo Fisher Scientific, Waltham, MA, USA). Proof of the enzymatic activity. Esterase turns acetoxymethyl calcein to calcein. Excitation 488 nm, emission 500–550 nm. In this study, the enzymatic activity was not evaluated.
3. Ethidium homodimer (EthD-1, red in Figure 4b,c; Thermo Fisher Scientific, Waltham, MA, USA). Nuclear stain that permeates through damaged cell membranes, thus cells with EthD-1 stained nuclei were evaluated as non-viable. Excitation 561 nm, emission 585–650 nm.

A laser confocal microscope Leica-SP8-TCS WLL-SMD-FLIM (Leica Microsystems CMS GmbH, Manheim, Germany) equipped with 63× W 1.2 NA objective was used for scanning the tissue slices obtained by PC and LC. The 405 nm diode laser (PicoQuant, Berlin, Germany) and the white light laser tuned to 488 and 561 nm were used for excitation (output of all adjusted to 20 μW at the sample position) and emission was detected on HyD detectors (420–475, 500–550, and 585–650 nm, respectively). To overcome bleed-through, images were acquired by line sequential scanning. The slices were scanned in four parallel planes along the Z-axis at the depth of 0, 12, 24, and 36 μm from the surface of the circular segment (Figure 4b,c) and/or from the internal (Figure 4c) (i.e., endothelial surface of the stripe, Figure 4b). For each PC or LC sample, at least three Z-stack were acquired. To assess the cell viability, the number of nuclei in both Hoechst and EthD-1 channel was counted using in-house developed semi-automatic mechanism written in the macro language of ImageJ (NIH, Bethesda, MD, USA). Briefly, Gaussian blur (0.5 μm) was applied to both channels, then binary masks for Hoechst and EthD-1 were created using the triangle threshold method, followed by automatic sum of particles on the binary mask (Figure 4b). The size (8–150 μm^2^) and circularity (0.2–1.0) of the recognized binary particles were conditions used for accepting them as cell nuclei. Finally, the post-thaw cell viability was calculated as the percentage of the sum of cells with EthD-1 (red) unstained nuclei from all cell nuclei found in the scans (Equation (1)). Viability was calculated separately for PC and LC.

(1) $$Viability\;\left[\%\right]=\frac{sum\;of\;cells\;with\;red\;unstained\;\;nuclei}{sum\;of\;all\;cells}\times 100$$

### 4.3. Statistical Evaluation 

Cell viability was assessed based on the following basic descriptive statistics: mean, standard deviation, and median. These were calculated for each group and are presented in Table 1, Table 2 and Table 3 and the box plots in Figure 1, Figure 2 and Figure 3 Detailed statistical evaluation and comparison of viability in individual groups and post-thaw times were performed in the Technology Center of the Czech Academy of Sciences. For evaluation of viability performed immediately after thawing the Shapiro–Wilk W test and Shapiro–Francia W test were used for the assessment of the normal distribution of data. Equality of variances was assessed using the Kruskal–Wallis equality of populations rank tests. Two sample t-tests with equal variances and one-way ANOVA test were used for a comparison of results in individual groups. For the assessment of viability changes in time global (omnibus) tests and post-hoc tests were used. In cases of unequal variances, the Greenhouse–Geisser (G–G) correction was performed. 

## 5. Conclusions

The method of viability assessment using fluorescence vital dyes and confocal microscopy seems to be a useful tool for the evaluation of the efficacy of freezing and thawing protocols of CVG. Our results showed immediate mean and median post-thaw viability higher than 70% in using both the slow and rapid thawing protocols after long-term storage in liquid nitrogen temperatures and good stability of results in short-term tissue culture. A statistically significant decline in the post-thaw cell viability through time was observed only in the slowly thawed grafts. The possible explanation for this might be the cell damage caused by the “solution effect”, which resulted in the delayed cell death. It is possible that similar events occur after graft transplantation, which can lead to the gentle reduction in its cellularity. The possible influence of this phenomenon on the immunogenicity of CVG should be the subject of further investigations. 

## Figures and Tables

**Table 1:** Immediate post-thaw cell viability in %.

	Slow Thawing-PC	Slow Thawing LC	Rapid Thawing PC	Rapid Thawing LC
**Mean**	87.70	74.5	85.5	81.2
**SD**	11.30	13.7	16.7	12.6
**Median**	91.03	79.0	94.0	80.0

**Table 2:** Post-thaw cell viability (%) after 24 and 48 h of culture–slow thawing protocol.

	PC 24 h Culture	LC 24 h Culture	PC 48 h Culture *	LC 48 h Culture
**Mean**	90.0	83.3	56.7	75.5
**SD**	5.9	20.9	23.7	15.2
**Median**	92.0	93.5	53.0	81.0

* Statistically significant decline of viability.

**Table 3:** Post-thaw cell viability (%) after 24 and 48 h of culture—rapid thawing protocol.

	PC 24 h Culture	LC 24 h Culture	PC 48 h Culture	LC 48 h culture
**Mean**	80.7	77.7	76.2	78.7
**SD**	22.6	14.6	15.5	15.3
**Median**	89.5	80.5	74.5	83.5

## Data Availability

Not applicable.

## Supplementary Materials

The following are available online at https://www.mdpi.com/article/10.3390/ijms221910653/s1.
